# A deep transfer learning radiomics nomogram using chest CT for differentiating anterior mediastinal cysts from low-grade thymomas

**DOI:** 10.3389/fonc.2026.1785331

**Published:** 2026-09-03

**Authors:** Li Zhao, Dengwang Zhao, Tong Zhou, Xueqing Sui, Pei Nie, Chongfeng Duan

**Affiliations:** 1 Department of Radiology, The Affiliated Hospital of Qingdao University, Qingdao, China; 2 Department of Nephrology, No. 971 Hospital of Chinese Navy, Qingdao, China

**Keywords:** anterior mediastinal cysts, chest CT, deep transfer learning, low-grade thymomas, radiomics

## Abstract

**Objectives:**

The objective of this study was to develop a nomogram by integrating clinical features, radiomics predictive results, and deep transfer learning (DTL) predictive results to differentiate between anterior mediastinal cysts (AMCs) and low-grade thymomas.

**Methods:**

The training set included 149 cases of AMCs and low-grade thymomas, whereas the test set included 49 cases of AMCs and low-grade thymomas. Contrast-enhanced chest CT images were used for analysis. A deep transfer learning radiomics (DTLR) nomogram was developed by integrating selected clinical features, radiomics predictive results, and DTL predictive results. Receiver operating characteristic (ROC) curves and decision curve analysis (DCA) curves were subsequently plotted.

**Results:**

This study identified clinical features, including maximum diameter, primary site, and relation to surroundings, to construct a clinical model. The DTLR nomogram demonstrated optimal predictive performance in the test set, achieving an area under the receiver operating characteristic curve (AUC) of 0.965, an accuracy of 0.857, a sensitivity of 0.895, and a specificity of 0.833. DCA demonstrated that DTLR is not optimal and is not significantly different from DTL. The DeLong test confirmed statistically significant differences in predictive performance between the DTLR nomogram and both the clinical model and the radiomics model, whereas no significant intermodal differences were observed among the other comparative approaches. DCA indicated that the predictive value of the DTLR nomogram was comparable to that of the DTL model. However, the DTLR nomogram demonstrated superior performance in the DeLong test, suggesting its potential as an effective clinical decision-support tool.

**Conclusions:**

Although further validation is needed before clinical implementation, the DTLR nomogram demonstrated favorable predictive performance and showed promise as a practical tool for clinical decision-making.

## Introduction

With the widespread use of chest computed tomography (CT) scans, the incidental discovery of anterior mediastinal masses has become increasingly common. Thymomas and anterior mediastinal cysts (AMCs), including thymic cysts and bronchogenic cysts, constitute the most common space-occupying lesions in the anterior mediastinum (1). Asymptomatic AMCs can be managed without surgery, whereas thymomas should be surgically resected as early as possible once they are diagnosed. Because many anterior mediastinal lesions lack specific imaging features, their differential diagnosis poses a new challenge for radiologists. Low-grade thymomas are typically spherical or oval. Owing to their shape, AMCs can be misdiagnosed as thymomatous lesions (2). Thymoma is the most prevalent neoplastic lesion among anterior mediastinal masses (3–5). According to the World Health Organization classification, thymic epithelial tumors are stratified into histologically distinct subtypes and are broadly categorized as either thymoma (including types A, AB, B1, B2, and B3) or thymic carcinoma (which encompasses neuroendocrine carcinomas) (6, 7). On the basis of tumor biological behavior and invasiveness, thymic epithelial tumors are categorized into low-risk thymomas (encompassing types A, AB, and B1), high-risk thymomas (including types B2 and B3), and thymic carcinomas (8). When an AMC has a CT density greater than 20 Hounsfield unit (HU) (9–11), its attenuation is similar to that of a low-grade thymoma, making the two entities difficult to distinguish. AMCs are frequently misdiagnosed as thymomas, resulting in nontherapeutic thymectomy rates ranging from 22% to 68% (12–14), which results in patients being subjected to unnecessary procedures and the consumption of significant healthcare resources. Given the proximity of these anterior mediastinal masses to the heart and major mediastinal vessels, invasive diagnostic approaches such as endoscopic needle biopsy carry substantial risks (15). Therefore, contrast-enhanced CT (CECT) is required for further evaluation. On CECT, high-density AMCs show no enhancement or only rim enhancement, whereas low-grade thymomas typically exhibit homogeneous mild enhancement. However, owing to hardening artifacts from major mediastinal vessels and thoracic bones, the CT values measured on enhanced mediastinal window scans are often inaccurate and sometimes even lower than those measured on non-contrast CT (NCCT). This inaccuracy can lead to preoperative misdiagnosis of these cysts as thymomas, resulting in unnecessary surgical interventions for some patients.

Radiomics (16–19) quantitatively and objectively predicts outcomes through the high-throughput extraction of textural parameters from medical images and the construction of predictive models. In deep learning (DL), convolutional neural networks (CNNs) are the most widely used architecture (20, 21). Although they are grounded in numerous real-world images, building an effective CNN model for medical research can be challenging because of the typically limited sample sizes. Therefore, much of the related research has focused on deep transfer learning (DTL). DTL (22–24) involves leveraging a model pretrained on a large-scale dataset (e.g., ImageNet), where it learns to recognize a wide array of fundamental image features. These features can serve as a foundational basis for other image-related tasks. Several studies have utilized radiomics or DTL to differentiate thymomas from AMCs using NCCT or CECT (25). However, to our knowledge, no study has yet employed a nomogram that integrates clinical, radiomics, and DTL features using CECT images to differentiate between low-grade thymomas with a maximum diameter of less than 5 cm and homogeneous density and AMCs.

In this study, we aimed to differentiate between low-grade thymomas with a maximum diameter of less than 5 cm and homogeneous density and AMCs using CECT images to improve preoperative diagnostic accuracy and assist in the formulation of clinical treatment strategies. In clinical practice, the prominent advantages of a nomogram are its intuitiveness and ability to clearly convey information. It offers a clear and intuitive method for representing complex mathematical relationships, thereby enabling the effective conveyance of information to nonspecialists and assisting in rapid clinical decision-making for estimating patient risk.

## Materials and methods

### Patients

This retrospective study was approved by the Institutional Review Board of the Affiliated Hospital of Qingdao University, with a waiver of informed consent, and was conducted in accordance with the relevant guidelines and regulations of the Declaration of Helsinki. This study retrospectively analyzed surgically resected anterior mediastinal masses at the hospital between January 2015 and June 2025. The inclusion criteria were as follows: (1) patients who underwent CECT examinations prior to surgery; (2) patients who had not received any treatment before surgery; (3) patients with pathologically confirmed low-grade thymoma, thymic cysts, or bronchogenic cysts after surgical resection; and (4) the presence of a solitary, homogeneous anterior mediastinal mass with a maximum diameter ranging from 0.5 cm to 5 cm. The exclusion criteria were as follows: (1) CT images with significant artifacts that compromised radiological evaluation and (2) the absence of a definitive pathological diagnosis or grading. In addition, clinical data, including age and sex, were collected.

### CT examination and CT feature analysis

All patients underwent the following scans: Siemens Sensation Cardiac 64-slice spiral CT instruments, GE Optima 620 CT, Toshiba Aquilion ONE 640-slice dynamic volume CT, GE Lightspeed 16-slice CT, or GE Brightspeed 16-slice CT. The standard scanning parameters were as follows: tube current: 250–350 mA, tube voltage: 120 kV, pitch: 0.9, slice thickness: 5 mm, and slice interval: 5 mm.

In the training and test sets, identical CT acquisition parameters were used. The chest CT scanning range extended from the thoracic inlet to the adrenal glands. All patients were scanned in the supine position following deep inspiration. Two thoracic radiologists, with 20 and 10 years of clinical experience, reached a consensus in analyzing the CECT imaging features. Imaging characteristics included maximum diameter, primary site, lesion edge, relation to surroundings, and lesion shape. Maximum diameter was the longest dimension measured on the maximum cross-sectional area of the lesion. Primary site was defined relative to the midline of the sternum and trachea. A lesion was classified as centrally located if its center was aligned with the midline of the trachea and sternum; otherwise, it was classified as non-central. Lesion edge was defined based on the smoothness of the lesion border. A lesion with a well-defined, sharp, and clearly demarcated contour was classified as smooth, whereas a lesion with a lobulated, spiculated, irregular, or ill-defined border was classified as non-smooth. The relation to surroundings was defined based on the interface between the lesion and the adjacent mediastinal pleura or lung margin. If the interface between the lesion and the mediastinal pleura or lung margin appeared as a straight line, it was classified as flat; if the interface exhibited bulging or convexity, it was classified as non-flat. Lesion shape was categorized as either regular or irregular. Round and oval configurations were defined as regular, whereas all other shapes, including lobulated or irregular forms, were classified as irregular.

### Image preprocessing and tumor segmentation

**Figure 1:**
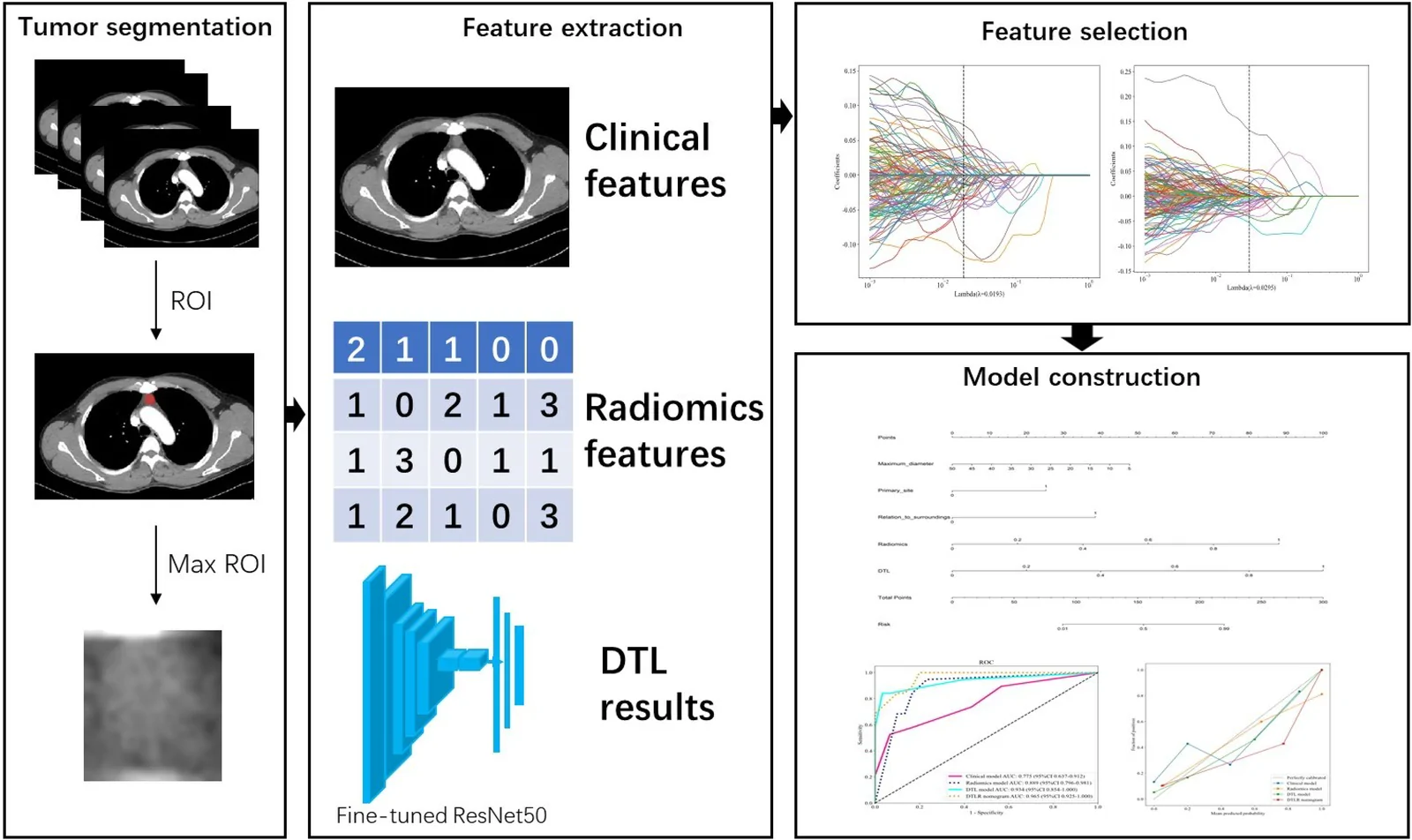
The workflow of the study. ROI, Region of interest; DTL, Deep transfer learning.

The workflow is shown in **Figure 1**.

All images were set to a consistent window width and window level (400 HU and 40 HU, respectively). All tumor slices were included for analysis, with each tumor represented by a three-dimensional region of interest (ROI). During the delineation of the ROIs along the tumor margins on the processed images, care was taken to avoid adjacent anatomical structures.

Image resampling enables voxel size standardization across diverse imaging datasets, ensuring direct comparability of data among different participants or study cohorts. To preserve scale and orientation invariance, all images were resampled to an isotropic voxel size of 1 × 1 × 1 mm³ (26). This spatial normalization ensures data consistency and facilitates the comparative analysis and integration of multisource data.

### Radiomics features extraction

Radiomics features were extracted using PyRadiomics package and divided into seven categories, including first-order features, shape features, Gray Level Co-occurrence Matrix (GLCM), Gray Level Run Length Matrix (GLRLM), Gray Level Size Zone Matrix (GLSZM), Gray Level Dependence Matrix (GLDM) and Neighboring Gray Tone Difference Matrix (NGTDM). Twenty CT images of low-grade thymomas and AMCs were randomly selected. Two thoracic radiologists with 10 and 20 years of clinical experience independently delineated the ROIs to calculate the intraclass correlation coefficients (ICCs) of the radiomics features. Features demonstrating high stability (ICC ≥ 0.8) were retained for subsequent analysis.

### Clinical and radiomics features selection

Clinical features were first evaluated through univariate regression analysis and subsequently refined using a multivariate regression model for final selection. These selected features were then incorporated into a clinical prediction model.

Radiomics features selection was conducted in a two-step process. First, the Spearman correlation coefficient was used for the initial screening. Correlation analysis was performed for each pair of features. If the correlation coefficient exceeded 0.9, the feature with superior predictive performance was retained on the basis of its predictive performance in preliminary modeling. Subsequently, least absolute shrinkage and selection operator (LASSO) regression analysis was used for feature dimensionality reduction and final refinement. Features were selected, and models were constructed separately for the arterial and venous phases. The predictive outputs from these phase-specific models were then integrated to construct a single, unified model, termed the radiomics model.

### Deep transfer learning

The workstation was equipped with an Intel i9-13900KF CPU and an NVIDIA RTX 4090 GPU with 24 GB GDDR6X video memory for DTL. To balance computational demands and hardware constraints, DTL was performed using two-dimensional regions of interest (2D ROIs) rather than three-dimensional regions of interest (3D ROIs). Specifically, a maximal 2D ROI was initially derived from the 3D ROI within axial image slices to facilitate subsequent feature analysis. Input images were then resized to a standardized dimension of 224 × 224 pixels. The pretrained CNN ResNet50 was utilized to support the subsequent analytical steps because ResNet50 provided stronger feature representation compared with shallow ResNet variants, while it incurred lower computational costs than deeper ResNet and Vision Transformers. The model was pre-trained on ImageNet with all convolutional layers fully fine-tuned. Cosine annealing learning rate decay and cross-entropy loss were employed. The validation set comprised 25% of the dataset. Training loss and accuracy curves were utilized as indicators to assess model convergence. Images were standardized using ImageNet’s RGB mean and standard deviation, with random horizontal flipping and random cropping as augmentation techniques. Batch Normalization served as the regularization approach for DTL. Hyperparameters were optimized via grid search. During the fine-tuning process, the batch size and the number of training epochs were set to 32 and 30, respectively. For the fine-tuning process, stochastic gradient descent (SGD) was employed as the optimization algorithm, with the learning rate set to 0.01, and the model was trained for 30 epochs. Training was halted if the training loss showed no reduction within 10 consecutive epochs.

### Model construction

The z-score standardization was conducted on all features before analysis. Clinical, radiomics, and DTL models were developed using the K-nearest neighbors (KNN) algorithm with selected features; subsequently, the deep transfer learning radiomics (DTLR)nomogram prediction model was constructed by integrating the selected clinical features and the outputs from the radiomics and DTL predictions. Comparative analyses were conducted among the clinical model, radiomics model, DTL model, and DTLR nomogram. The model evaluation incorporated both visual analytical tools and quantitative performance metrics. The visual assessment included receiver operating characteristic (ROC) curves, calibration plots, and decision curve analysis (DCA) curves. Calibration curves were used to evaluate prediction accuracy, while DCA was employed to assess clinical utility. The performance metrics included the area under the receiver operating characteristic curve (AUC), specificity, accuracy, and sensitivity.

### Statistical analysis

Data analysis was performed using Python software. Categorical and continuous variables were compared between the two groups using the chi-square test and t test, respectively. Univariate and multivariate logistic regression analyses were conducted to identify significant clinical features. The DeLong test was employed to compare differences in AUCs among models, while the Hosmer–Lemeshow test was used to evaluate the goodness-of-fit. The significance level was set at p < 0.05. The One-Key AI platform (http://www.medai.icu) was used to process the data, and the code is available at https://gitee.com/wangqingbaidu/OnekeyCompo?_from=gitee_search.

## Results

A total of 222 AMC cases and 160 thymoma cases were collected from our institution. After screening against exclusion criteria 1 and 2, which led to the exclusion of 94 and 90 patients, respectively, a final cohort of 198 patients was included in the study, including 83 cases of thymoma and 115 cases of AMC. The training set and test set were divided randomly. The training set consisted of 149 cases, among which there were 64 thymomas and 85 AMCs, while the test set contained 49 cases with 19 thymomas and 30 AMCs. There was no statistically significant difference between two sets in each clinical feature (**Supplementary Table 1**).

**Figure 2:**
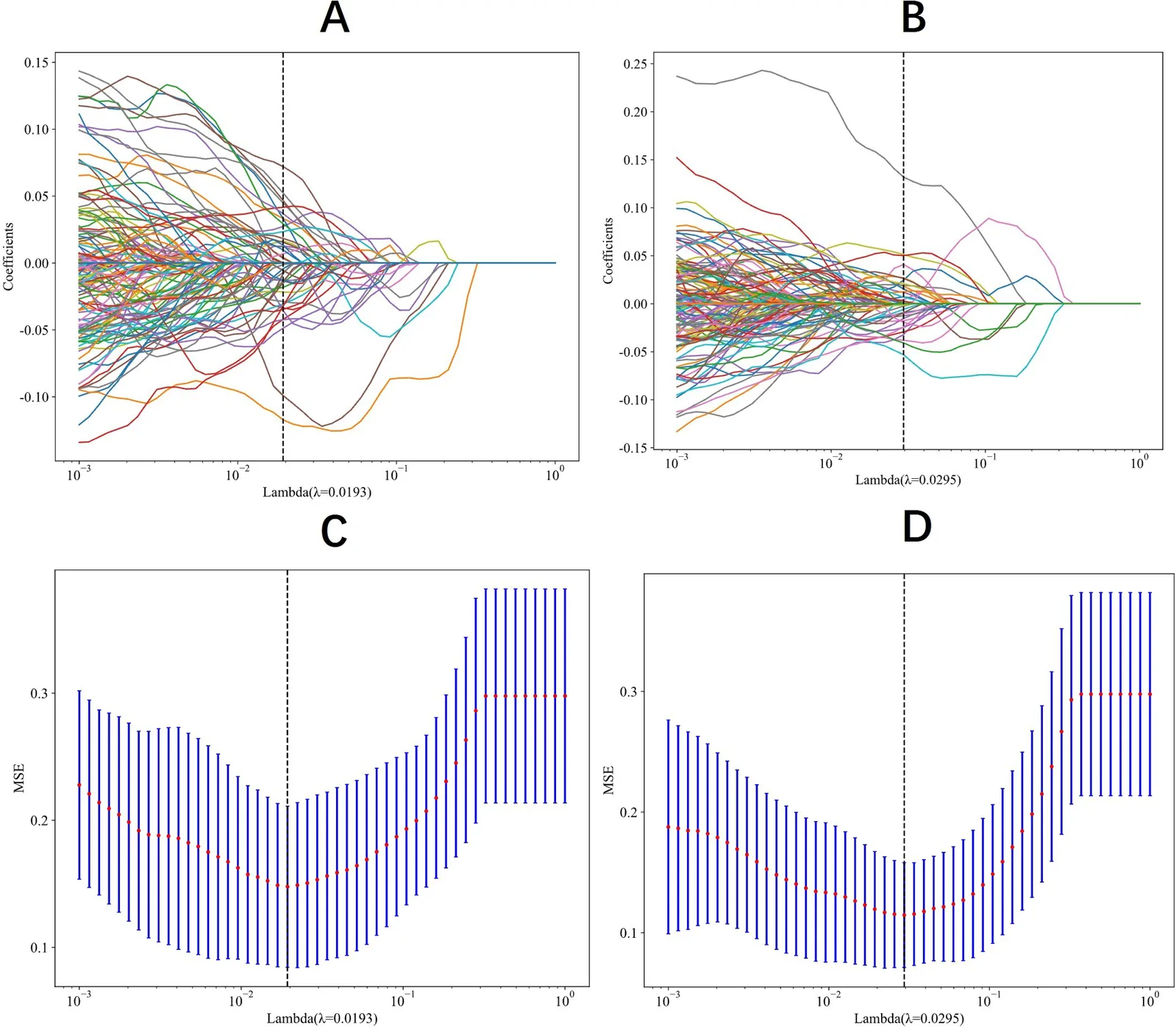
LASSO regression in radiomics feature selection [**(A, C)** arterial phase; **(B, D)** venous phase]. **(A, B)** The x axis represents the λ value, and the y axis represents the coefficients of features. Each colored line corresponds to one feature. As λ increases, more coefficients shrink toward zero. The optimal λ was the vertical dashed line selected by cross-validation. **(C, D)** Mean squared error (MSE) curve for LASSO regression. The x axis represents the regularization parameter λ, and the y axis represents MSE. The optimal λ was the vertical dashed line selected by cross-validation.

**Figure 3:**
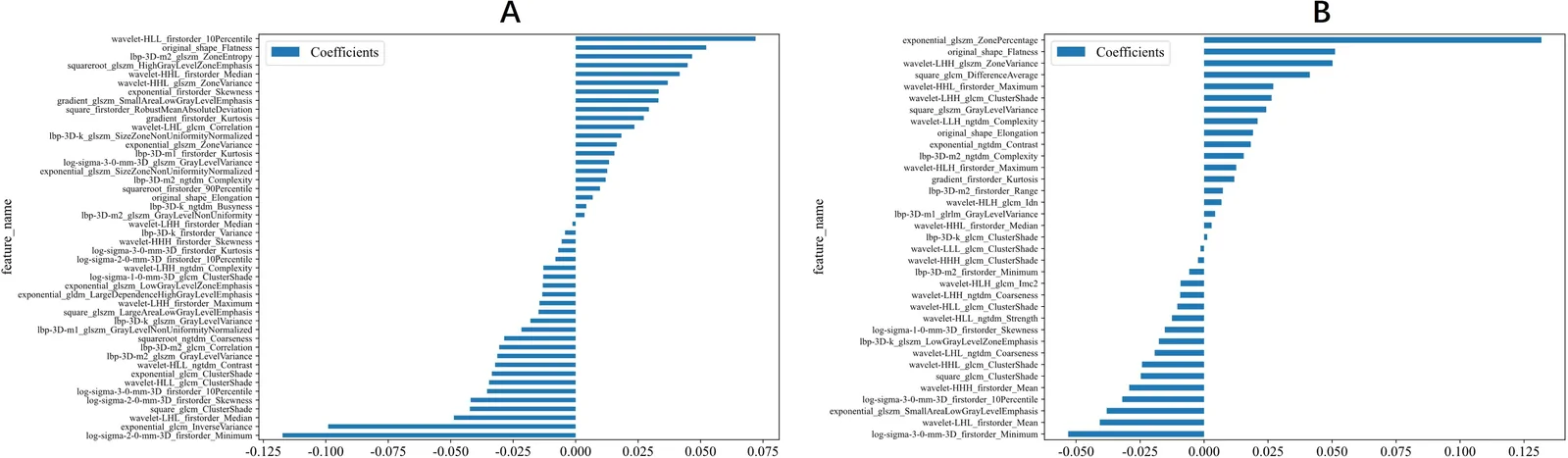
The selected radiomics features with the correlation coefficient [**(A)** arterial phase; **(B)** venous phase]. The x axis represents the value of correlation coefficients, and the y axis represents the selected features. The length of each blue bar corresponds to the value of respective feature.

**Table 1** presents the results of the univariate and multivariate regression analyses. The selected features used to construct the clinical model included the maximum diameter, primary site, and relation to surroundings because their p values were all less than 0.05 in univariate and multivariate regression analyses. A total of 1834 radiomics features were extracted from each ROI. After ICC analysis, Spearman analysis and LASSO regression were performed; 46 features were retained in the arterial phase and 35 features were retained in the venous phase, and the λ value obtained from LASSO regression was 0.0193 for the arterial phase and 0.0295 for the venous phase. **Figures 2A, B** show that more coefficients shrink toward zero as λ increases. The optimal λ was the vertical dashed line selected by cross-validation. **Figures 2C, D** show the mean squared error curve for LASSO regression. **Figure 3** shows the names and coefficient values of the selected radiomics features. The x axis represents the value of correlation coefficients, and the y axis represents the selected features. The length of each blue bar corresponds to the value of respective feature. **Table 2** shows the results of the clinical, radiomics, DTL and DTLR nomogram models. DTLR had the highest AUC of 0.996, with an accuracy of 0.966, a sensitivity of 0.953, and a specificity of 0.976 in the training set. DTLR had the highest AUC of 0.965, with an accuracy of 0.857, a sensitivity of 0.895, and a specificity of 0.833 in the test set.

**Table 1.** The results of univariate and multivariate regression analysis in clinical features.

Feature name	univariate regression analysis		Multivariate regression analysis	
	OR	P	OR	P
Gender	0.702	0.015	0.829	0.283
Age	1.218	0.172	-	–
Maximum diameter	2.876	< 0.001	2.214	< 0.001
Primary site	1.773	< 0.001	1.624	0.009
Lesion edge	1.716	0.001	1.372	0.223
Relation to surroundings	2.485	< 0.001	2.137	< 0.001
Lesion shape	1.583	0.007	0.916	0.752
Clinical symptoms	0.801	0.132	-	–

OR Odds Ratio

**Table 2.** The results of clinical model, radiomics model, DTL model and DTLR nomogram.

	Accuracy	AUC (95% CI)	Sensitivity	Specificity	PPV	NPV
Training set						
Clinical model	0.805	0.898 (0.8520 - 0.9445)	0.625	0.941	0.889	0.769
Radiomics model	0.940	0.968 (0.9384 - 0.9984)	0.891	0.976	0.966	0.922
DTL model	0.906	0.972 (0.9519 - 0.9915)	0.828	0.965	0.946	0.882
DTLR nomogram	0.966	0.996 (0.9903 - 1.0000)	0.953	0.976	0.968	0.965
Test set						
Clinical model	0.694	0.775 (0.6369 - 0.9122)	0.211	1.000	1.000	0.667
Radiomics model	0.837	0.889 (0.7959 - 0.9813)	0.842	0.833	0.762	0.893
DTL model	0.837	0.934 (0.8539 - 1.0000)	0.579	1.000	1.000	0.789
DTLR nomogram	0.857	0.965 (0.9248 - 1.0000)	0.895	0.833	0.773	0.926

DTL deep transfer learning, DTLR deep transfer learning radiomics, AUC area under the receiver operating characteristic curve, CI confidence interval, PPV positive predictive value, NPV negative predictive value

**Figure 4:**
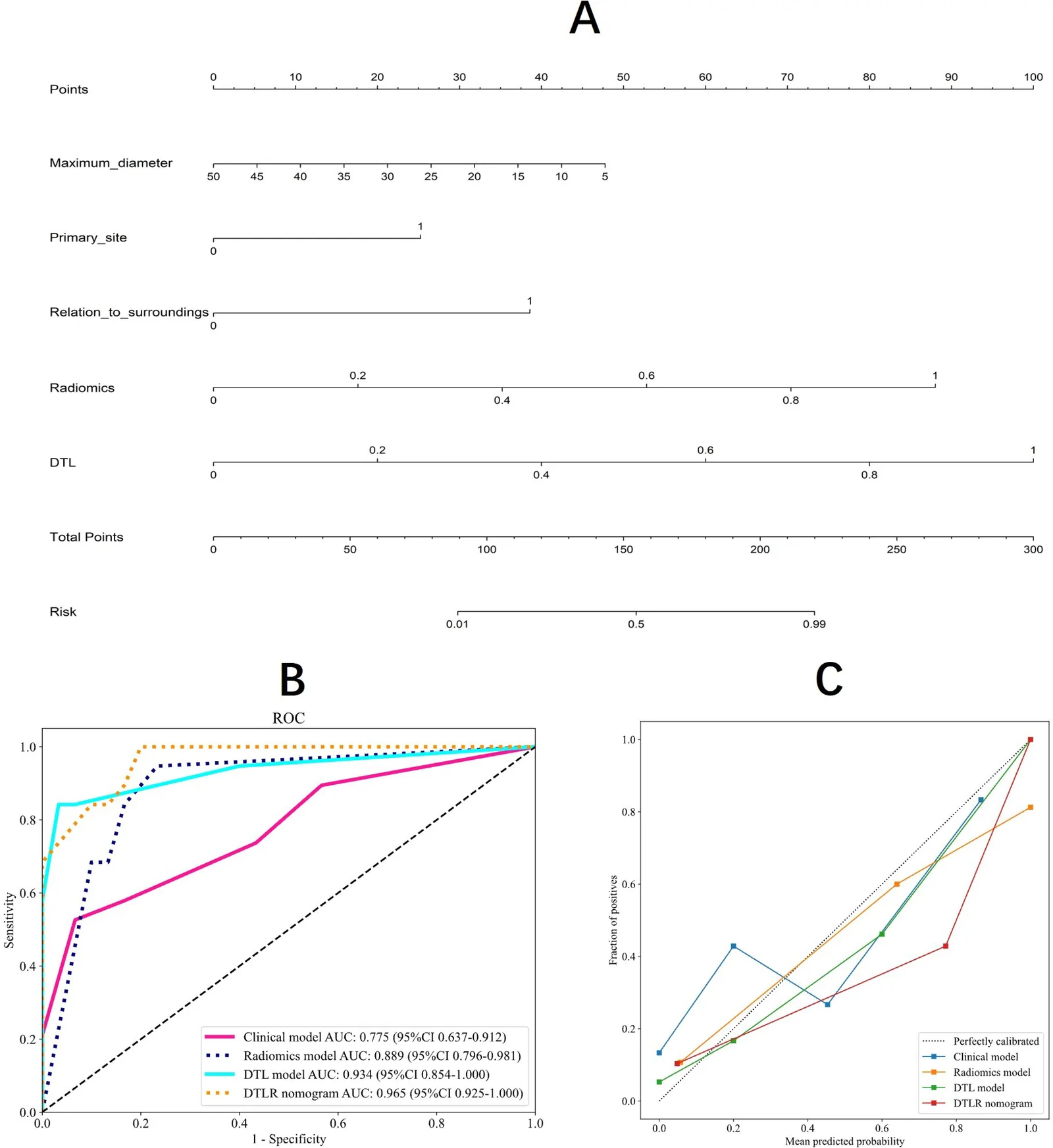
The DTLR nomogram included the clinical features of maximum diameter, primary site, relation to surroundings, Radiomics score and DTL score **(A)**. In the nomogram, maximum diameter, radiomics and DTL variables are pointed by their values, while primary site and relation to surroundings are pointed according to real conditions (0 represents central site and flat;1 represents non-central site and non-flat). The summed total points determine the risk of thymoma. The ROC curves **(B)** and calibration curves **(C)** of the clinical model, radiomics model, DTL model and DTLR nomogram. **(B)** The ROC curves of the four models in test set. Curves with different colors represent different models. DTLR (yellow dashed line) had the highest AUC of 0.965. **(C)** The calibration curves of the four models. The dashed line represents perfect calibration, while solid lines in different colors represent the calibration performance of four models. DTL, Deep transfer learning; DTLR, Deep transfer learning radiomics.

The DTLR nomogram is presented in **Figure 4A**. In the nomogram, maximum diameter, radiomics and DTL variables are pointed by their values, while primary site and relation to surroundings are pointed according to real conditions (0 represents central site and flat;1 represents non-central site and non-flat). The summed total points determine the risk of thymoma. The ROC and calibration curves of the four models are shown in **Figures 4B, C**. The AUCs of the clinical, radiomics, DTL, and DTLR nomogram models were compared using the DeLong test (**Table 3**), and the DTLR predictive model demonstrated significant differences when compared separately with the clinical model and the radiomics model. There was no significant difference in pairwise comparisons among other models.

**Table 3.** The results of DeLong test.

	Clinical model	Radiomics model	DTL model	DTLR nomogram
Clinical model	–	0.1823	0.0546	0.0074
Radiomics model	0.1823	–	0.4338	0.0225
DTL model	0.0546	0.4338	–	0.3756
DTLR nomogram	0.0074	0.0225	0.3756	–

DTL deep transfer learning, DTLR deep transfer learning radiomics

**Figure 5:**
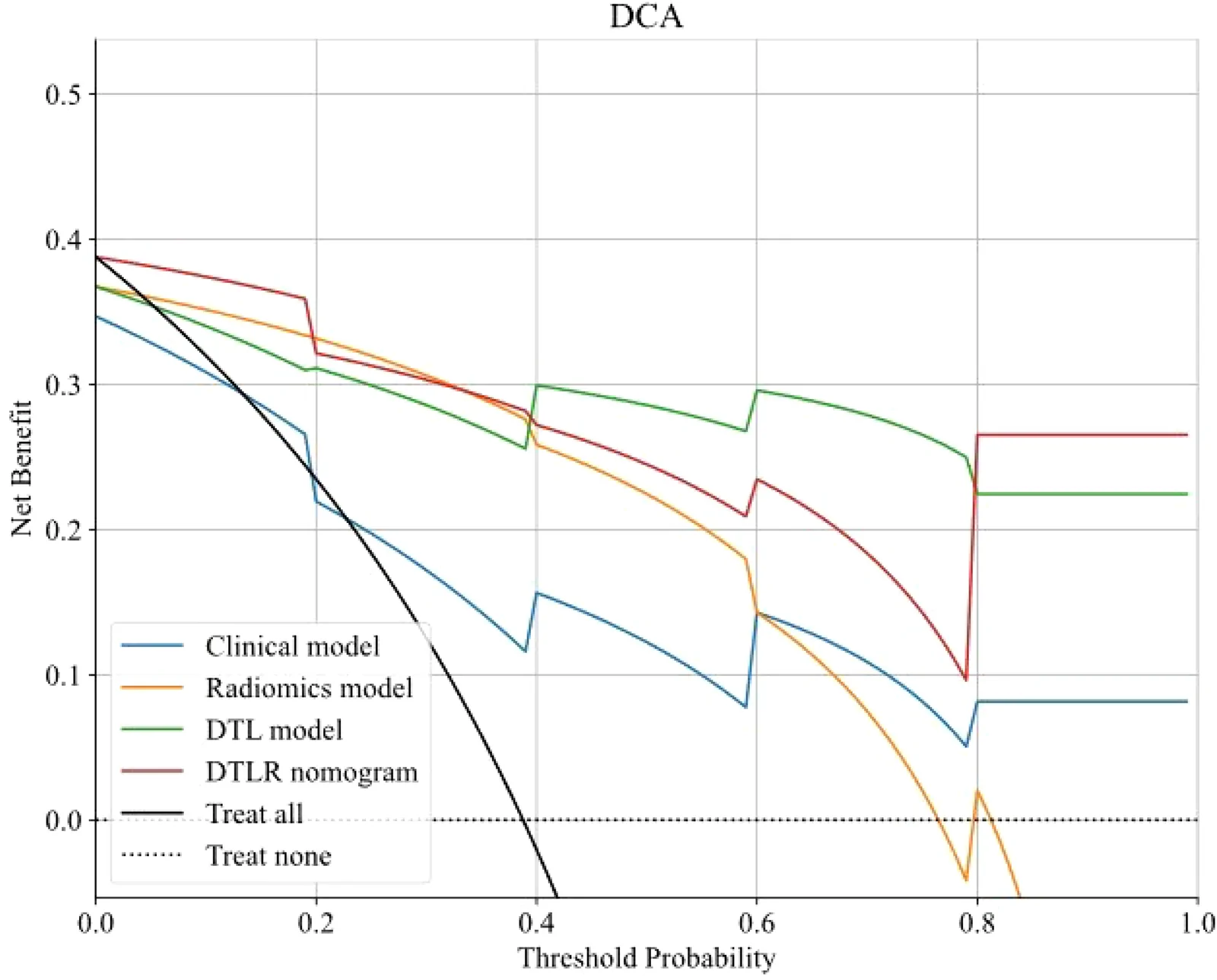
DCA curves of the clinical model, radiomics model, DTL model and DTLR nomogram. The x axis represents the threshold probability, and the y axis represents the net benefit. Solid lines with different colors represent different models. There is no significant difference between DTLR nomogram and DTL model in DCA. They both perform better than clinical and radiomics model in DCA. DTL, deep transfer learning; DTLR, deep transfer learning radiomics.

The Hosmer–Lemeshow test results for the clinical, radiomics, DTL, and DTLR nomogram were 0.464, <0.01, 1, and 0.838, respectively. The DCA shown in **Figure 5** indicates that the DTLR model does not yield the greatest net benefit. Furthermore, an intersection between the DCA curves of DTLR and DTL suggests no statistically significant difference between the two models.

## Discussion

In this study, we developed a nomogram that integrated clinical features, radiomics, and DTL to differentiate low-grade thymomas (maximum diameter <5 cm and homogeneous density) and AMCs using CECT images. The DTLR nomogram achieved an AUC of 0.965 in the test set, with an accuracy of 0.857, sensitivity of 0.895, and specificity of 0.833.

Radiomics and DL has been extensively used in differential diagnosis of anterior mediastinal lesions (27–32). Liu et al. (28) extracted histogram parameters from unenhanced CT images and compared a tumor group (thymoma and lymphoma) with a nontumor group (hyperplasia and cysts). ROC analysis demonstrated that a higher median value served as an independent predictor for distinguishing tumors from non-tumors, with an AUC of 0.785. Shang et al. (29) utilized NCCT images to develop a radiomics model and a combined model that integrated clinical characteristics, subjective CT findings, and radiomics features for discriminating between AMCs and thymomas. Liu et al. (30) developed an integrated model combining a radiomics signature with independent clinical factors using both CECT and NCCT images. The combined model based on CECT demonstrated superior diagnostic performance, with AUC values of 0.941 and 0.938 in the training and test cohorts, respectively. This combined model significantly outperformed both the model based on NCCT and the standalone radiomics model derived from CECT. Yang et al. (31) developed a combined model by integrating radiomics features with clinic radiological characteristics and incorporating a DTL model. This integrated model demonstrated strong diagnostic performance, with AUC values of 0.933 and 0.945 in the internal and external validation cohorts, respectively. Yang et al. (32) integrated radiomics and clinicopathological data to conduct a comprehensive analysis of DTL networks with varying architectures and dimensionalities. Their findings demonstrate that multidimensional DTL models can serve as noninvasive diagnostic tools with high sensitivity and specificity for differentiating thymomas from thymic cysts.

Consistent with previous studies, this study used enhanced images from 198 cases of AMCs and low-grade thymomas, providing a relatively large sample size and imaging dataset. Both radiomics and DL are fundamentally grounded in artificial intelligence and big data analytics, for which the utilization of larger sample sizes facilitates the derivation of more reliable and robust diagnostic outcomes. Furthermore, this research has achieved several key advancements. Earlier radiomics research was restricted to a small set of radiomics features, such as 13, 73, 180. A total of 1834 radiomics features were extracted in the present study. Compared with prior studies using limited radiomics features, this study extracted high-dimensional features to fully capture subtle intralesional heterogeneity invisible on conventional CT. The expanded feature pool avoids key biomarker omission, enables rigorous feature screening, and allows machine learning models to learn lesion differences sufficiently, improving model stability, generalization and diagnostic reliability for differentiating. Furthermore, no prior research had constructed an integrated model combining clinical characteristics, radiomics and DTL signatures. Thus, the present study represents a novel exploratory attempt. To the best of our knowledge, this study represents the first development and validation of a DTLR-based discriminative model utilizing CECT images for distinguishing AMCs from low-grade thymomas. The integrated model realizes complementary strengths by fusing three types of information. Clinical features provide baseline risk data, radiomics quantifies subtle intralesional heterogeneity, and DTL extracts high-level imaging representations. Multi-source fusion improves discrimination and calibration, elevates clinical net benefit, alleviates overfitting, and delivers better cross-cohort generalization than single-modality models, balancing interpretability and predictive performance.

The nomogram (**Figure 4A**) integrates clinical, radiomics, and DTL-derived features for individualized risk estimation. The nomogram provides individualized risk estimates to assist in preoperative differentiation between AMCs and low-grade thymomas. The score components include the maximum diameter, primary site, relation to surroundings, radiomics and DTL. In the test set, the DTLR model achieved the highest AUC value and was superior to the other two models according to the DeLong test. Although no statistically significant difference was observed between the DTLR and DTL models, the DTLR model demonstrated a higher AUC. Clinical utility was further evaluated using DCA. The DTLR model showed good calibration, while DCA indicated comparable net benefit between DTLR and DTL.

Although the DTL model achieved strong standalone performance, the DTLR nomogram should be interpreted in the context of clinical decision system rather than pure performance optimization. In clinical practice, the value of models is not determined solely by incremental improvements in discrimination metrics, but also by their ability to integrate complementary information in a clinically meaningful and interpretable manner. DTL captures high-level imaging representations through data driven learning but lacks explicit clinical semantics. In contrast, radiomics provides handcrafted quantitative descriptors reflecting tumor intensity and heterogeneity, while clinical variables offer patient and lesion specific context. Accordingly, the DTLR nomogram represented a multimodal integration strategy that better aligned with clinical reasoning, where imaging and clinical information were jointly considered. From this perspective, the purpose of the DTLR nomogram was not necessarily to outperform the DTL model in terms of AUC or net benefit, but to enhance the interpretability and comprehensiveness of decision support. The lack of clear superiority in DCA did not imply redundancy, but might reflect the already strong baseline performance of the DTL model, leaving limited room for further improvement at the population level. Moreover, DCA evaluated average clinical net benefit and might not fully capture potential advantages in specific clinical subgroups or decision thresholds. Therefore, model comparison should consider not only discrimination performance, but also interpretability and clinical integration potential. Overall, the DTLR nomogram should be viewed as a step toward multimodal decision support rather than a purely performance driven enhancement. Further multicenter prospective validation is needed to determine its clinical utility across diverse populations and imaging settings.

Several limitations of this study should be acknowledged. First, this was a retrospective single center study, which may have introduced selection bias and limited the generalizability of the proposed model. Because all patients were recruited from a single center, the imaging characteristics and disease spectrum may not fully reflect those encountered in other institutions or broader clinical settings. In addition, only surgically resected and pathologically confirmed cases were included, whereas conservatively managed AMCs without histopathological confirmation were excluded. This inclusion criterion may have further contributed to selection bias by preferentially enrolling lesions requiring surgical intervention, thereby limiting the applicability of the model to the general population with anterior mediastinal lesions. Second, no external validation cohort from other institutions was available. Therefore, the robustness and transportability of the model across different scanners, imaging protocols, and patient populations remain uncertain. Multicenter prospective studies with external validation cohorts are warranted to further assess the reproducibility and clinical applicability of the proposed model. Third, the relatively small size of the test cohort (n = 49) may have affected the stability of the estimated diagnostic performance and reduced the statistical power for model comparison. Although multiple strategies were applied to mitigate overfitting, including using ICC analysis, Spearman correlation filtering, and LASSO regression, as well as comprehensive evaluation using calibration analysis, the DeLong test, and DCA, the possibility of performance overestimation cannot be fully excluded. Overall, these limitations suggest that further validation in large scale, multicenter, prospective cohorts is required before clinical implementation.

## Conclusion

In this study, a DTLR nomogram utilizing CECT images was developed to differentiate AMCs from low grade thymomas. Although further validation is required prior to clinical implementation, the model demonstrated favorable predictive performance and showed potential as a practical tool for clinical decision-making. Subsequent studies are required to conduct large-scale external validation based on prospective multicenter cohorts.

## Data Availability

The raw data supporting the conclusions of this article will be made available by the authors, without undue reservation.
