# Supplementary material for: A deep transfer learning radiomics nomogram using chest CT for differentiating anterior mediastinal cysts from low-grade thymomas
Source: Front Oncol. 2026 Sep 3;16:1785331. doi: 10.3389/fonc.2026.1785331 (PMC13581725; doi:10.3389/fonc.2026.1785331)
Supplement: Supplementary file 1 [file Table1.docx]

Supplementary Table 1. Comparison of Clinical features Between Training and Test Sets

| Clinical features | p value |
| --- | --- |
| Age | 0.417 |
| Maximum diameter | 0.322 |
| Gender | 0.920 |
| Primary site | 0.578 |
| Lesion edge | 0.351 |
| Relation to surroundings | 0.245 |
| Lesion shape | 0.709 |
| Clinical symptoms | 0.930 |
